# Exposure of an obstructed splenic artery aneurysm stent into the duodenum

**DOI:** 10.1055/a-2582-4085

**Published:** 2025-05-06

**Authors:** Yutaka Ogikubo, Shun Osumi, Shosuke Hosaka, Kazushi Fukagawa, Satoshi Ono

**Affiliations:** 1 542784 Gastroenterology and Gastrointestinal Endoscopy, Tokyo Metropolitan Geriatric Hospital and Institute of Gerontology Hospital, Tokyo, Japan

A 76-year-old man with a history of splenic artery aneurysm and celiac artery aneurysm was referred for a screening esophagogastroduodenoscopy (EGD). An endovascular stent had been placed for the splenic artery aneurysm 5 years previously ( Fig. 1 ), and coiling had been performed in the stent lumen 4 months previously 1 ( Fig. 2 ).

**Fig. 1:**
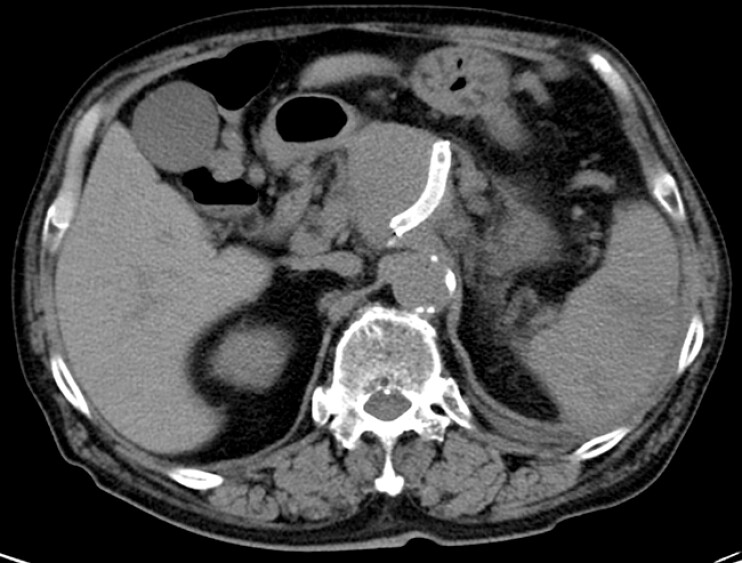
Computed tomography image showing an endovascular stent that had been placed in a splenic artery aneurysm.

**Fig. 2:**
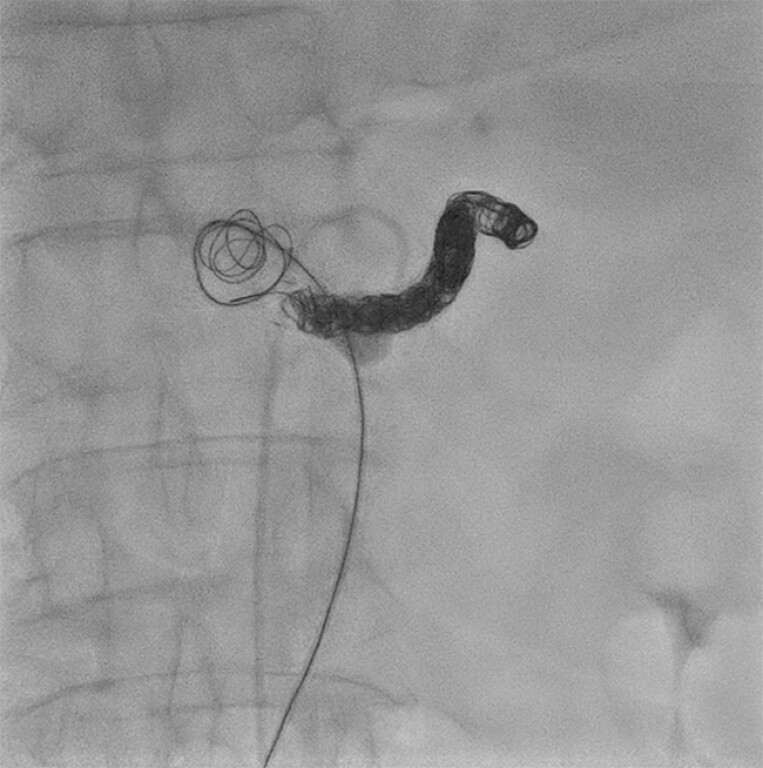
Fluoroscopic image showing a coil within the stent lumen.

EGD revealed exposure of a metal mesh in the duodenum, which was thought to be the endovascular stent that had been placed in a splenic artery aneurysm ( Fig. 3 ); no obvious bleeding was observed. Contrast-enhanced computed tomography was performed, but blood flow within the stent could not be evaluated owing to artifacts caused by the stent, and detailed observation was also difficult with transabdominal ultrasonography 2 . Therefore, endoscopic ultrasonography (EUS) was performed from within the stomach, taking care not to directly affect the exposed stent, and we were able to evaluate the stent through the metal mesh, with acoustic shadow but no obvious blood flow seen in the lumen on Doppler echo 3 ( Fig. 4 ; Video 1 ).

**Fig. 3:**
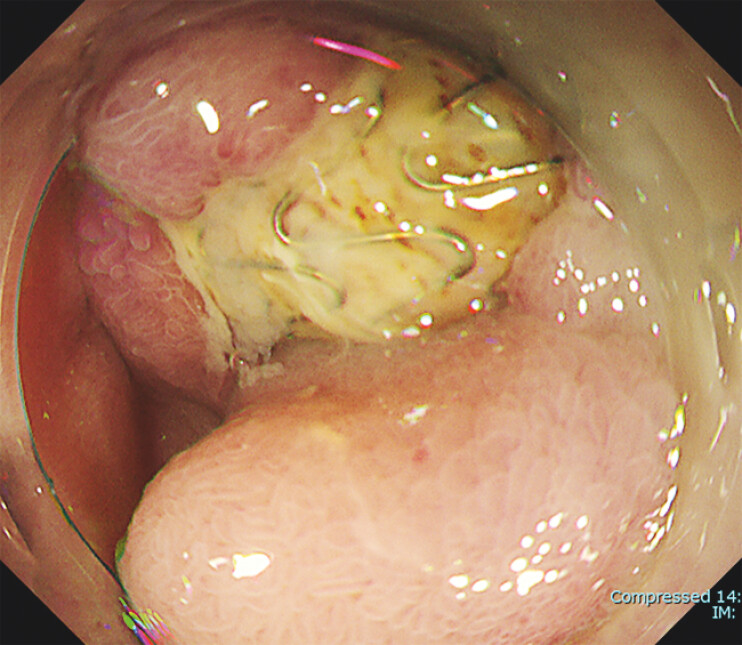
Endoscopic image showing a metal mesh exposed in the duodenum.

**Fig. 4:**
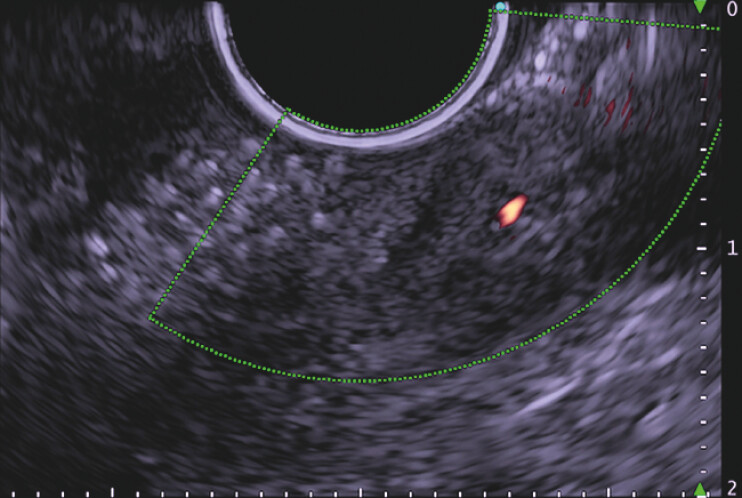
Endoscopic ultrasonography image showing no obvious blood flow in the stent lumen.

Esophagogastroduodenoscopy shows an exposed metal mesh in the duodenum, which was thought to be an endovascular stent placed in a splenic artery aneurysm; the stent was evaluated with endoscopic ultrasonography through the metal mesh, with acoustic shadow but no evidence of luminal blood flow on Doppler echo.Video 1

Surgical removal of the exposed stent was considered, but the patient was judged to be unsuitable for this owing to his severe co-morbidities. Instead, observation was chosen because of the fact that he had been able to manage oral intake without any problems.

Endoscopy_UCTN_Code_CCL_1AB_2AZ_3AZ

## Notes

Endoscopy E-Videos https://eref.thieme.de/e-videos
*E-Videos* is an open access online section of the journal *Endoscopy* , reporting on interesting cases and new techniques in gastroenterological endoscopy. All papers include a high-quality video and are published with a Creative Commons CC-BY license. Endoscopy E-Videos qualify for HINARI discounts and waivers and eligibility is automatically checked during the submission process. We grant 100% waivers to articles whose corresponding authors are based in Group A countries and 50% waivers to those who are based in Group B countries as classified by Research4Life (see: https://www.research4life.org/access/eligibility/ ). This section has its own submission website at https://mc.manuscriptcentral.com/e-videos .
